# Slice-Guided Components Detection and Spatial Semantics Acquisition of Indoor Point Clouds

**DOI:** 10.3390/s22031121

**Published:** 2022-02-01

**Authors:** Lijuan Wang, Yinghui Wang

**Affiliations:** 1 School of Electronic Information Engineering, Xi’an Technological University, Xi’an 710021, China; wanglijuan@xatu.edu.cn; 2 School of Artificial Intelligence and Computer Science, Jiangnan University, Wuxi 214122, China

**Keywords:** indoor scene components, point clouds, slices, spatial relationship, ontology

## Abstract

Extracting indoor scene components (i.e., the meaningful parts of indoor objects) and obtaining their spatial relationships (e.g., adjacent, in the left of, etc.) is crucial for scene reconstruction and understanding. At present, the detection of indoor scene components with complex shapes is still challenging. To fix the problem, a simple yet powerful slice-guided algorithm is proposed. The key insight is that slices of indoor scene components always have similar profiles no matter if the components are simple-shaped or complex-shaped. Specifically, we sliced the indoor scene model into many layers and transformed each slice into a set of two-dimensional (2D) profiles by resampling. After that, we clustered 2D profiles from neighbor slices into different components on the base of spatial proximity and similarity. To acquire the spatial relationships between indoor scene components, an ontology was constructed to model the commonsense knowledge about the semantics of indoor scene components and their spatial relationships. Then the spatial semantics of the relationships between indoor scene components were inferred and a semantic graph of spatial relationship (SGSR) was yielded to represent them. The experimental results demonstrate that our method can effectively detect complex-shaped indoor scene components. The spatial relationships between indoor components can be exactly acquired as well.

## 1. Introduction

Components of indoor scenes are meaningful parts of indoor objects. Detecting the indoor scene components and acquiring their spatial relationships (e.g., adjacent, in the right of, in the left of, etc.) is one of the most important research problems in the computer vision and graphics community. As pointed out in many studies [1,2,3], the acquirement of indoor scene components and their spatial relationships will benefit many computer vision works such as indoor scene reconstruction and indoor scene understanding [4,5].

There are two main difficulties that arise during the detection of indoor scene components and their spatial relationships: (1) indoor scene components often have varied shapes and complex three-dimensional (3D) geometry. Moreover, the indoor scene components occlude each other. Thus, it is challenging to detect complex-shaped indoor scene components from point clouds; (2) due to the diverse internal structures of indoor objects and the messy arrangement of indoor objects, the spatial relationships between indoor scene components are complex, which makes it difficult to extract the spatial relationships between indoor scene components.

Most of the approaches [6,7,8,9,10,11,12] for the detection of indoor scene components concentrate on using primitive shapes (e.g., planes, cylinders, spheres, cuboids, etc.) to approximate the components and exploit 3D primitive shape segmentation algorithms such as Hough transforming [13,14] and Random Sample Consensus (RANSAC) [15,16] to detect indoor scene components. In these approaches, the primitive shape features of indoor components are always pre-assumed, which is not suitable for complex-shaped indoor scene components.

Many methods [17,18,19] transform the scattered point clouds into 3D voxel grids and use spatial connectivity and geometric features to segment the indoor scene models. However, due to sparsity of the point clouds, the voxel grids may have empty voxels which leads to redundant computations. Moreover, it is difficult to select the appropriate resolution to accurately segment the components and preserve the boundaries due to the different scales of objects in the indoor scene model and the non-uniform point cloud density. 

With the availability of large 3D datasets and the popularity of machine learning techniques, some data-driven segmentation methods [20,21,22,23,24,25,26,27] have been proposed for indoor scene components. In previous data-driven methods [20,21,27], indoor scene models are first segmented. Then the segmented results of the indoor scenes are classified into different components based on handcrafted features by machine-learning techniques, e.g., conditional random field (CRF), support vector machine (SVM) and so on. Motivated by directly learning features from input point clouds, the deep neural network has recently been exploited. Qi et al. [22] designed a novel type of neural network (PointNet) to provide a unified architecture for feature classification directly from point clouds. On the architecture, the labelling of components of objects is performed. Followed PoinNet, other deep neural networks have been proposed, such as PointNet++ [28], the deep part induction network [23], the regularized graph convolutional neural network (RCGNN) [25], semantic part decomposition network [29] and so on. Although progress in detecting complex-shaped components is impressive, these methods are still inferior when it comes to discovering new components whose types are not covered in the training sets. 

There are also other methods. Balado et al. [30] proposed a method to detect floor elements based on relative distances. In the references [31,32], surface patches of indoor scene models were merged into components according to the consistency of their local convexity or non-local geometric signature. Due to poor connectivity caused by missing parts and outliers of point clouds, convexity-based methods are not reliable for detecting the indoor scene components. The detection of complex-shaped indoor scene components is still challenging. 

The extraction of the spatial relationships between indoor scene components lays a foundation for understanding the indoor scene in a way similar to the way that humans perceive the environment. Many methods [33,34,35] have been proposed to extract spatial relationships from scene images. In contrast with the spatial relationships in images, the spatial relationships in 3D point clouds are more complex [36,37], and the extraction of them is more challenging. 

Recently, a few methods [38,39] have been proposed to extract spatial relationships from indoor point clouds based on machine learning techniques such as SVM and latent max-margin learning. However, it is difficult to build up a fixed parameter model for training due to the complexity of 3D spatial relationships. To fix the problem, Wald et al. [40] recently tried to use deep learning techniques to train and predict spatial relations. The deep learning-based method showed prospects in the extraction of certain spatial relationships. However, spatial relationships in 3D space are complex. It is difficult to obtain salient features between different spatial relationships and to effectively divide the spatial relationships into different categories based on the feature.

On the other hand, some approaches [41,42,43,44] have been proposed to extract spatial relationships from indoor point clouds based on prior spatial knowledge. For example, Zender et al. [42] presented an ontology to encode the spatial and functional knowledge of typical indoor environments. Suchan and Bhatt [43] adopted prior knowledge to extract commonsense spatio-temporal relations. Most existing knowledge-based methods have aimed to provide root navigation for indoor robots or model specific interactions between human and indoor objects. They mainly focused on the inter-object or human-centric spatial relationships. As a smaller-grained element of scenes, spatial relationships between the indoor scene components are also affected by the structure of indoor objects. Accordingly, it is more difficult to detect the spatial relationships between indoor components.

In this paper, we present a framework to segment out the indoor scene components and detect their spatial relationships. Our method is based on a slice strategy. We are inspired by the methods in [45,46,47], where components of complex-shaped indoor objects were segmented based on the similarity of 2D profiles. Furthermore, our kernel insight lies in two points: (1) Slices of indoor scene components always have spatial proximity and similar profiles no matter if the components are simply or complex shaped. (2) The spatial topological relationships between indoor scene components can be effectively preserved by slicing the indoor scene layer by layer.

We use the slice strategy to obtain many slices of indoor scene models and convert each slice into a set of profiles, then merge the profiles of neighbor slices progressively into different components based on spatial proximity and similarity. Next, we geometrically establish relationships between the detected indoor scene components on the base of two geometric distances. Meanwhile, an ontology is built up to model the semantic knowledge about the spatial relationships between indoor scene components. The geometrically correlated indoor scene components are loaded to populate the ontology. Finally, the spatial semantics of the relationships are thereby inferred, and a semantic graph of spatial relationship (SGSR) is yielded to organize the indoor scene components and their spatial relationships. 

The contributions of this paper can be summarized as follows:

(1) We propose a slice-guided algorithm to detect complex-shaped indoor scene components from point clouds. The detected components are faithful to the meaningful parts of indoor objects;
(2) We present a framework for modelling indoor scene components and their spatial relationship structure, which lays a foundation for the detection of following objects, semantic analysis, and understanding of indoor scenes.

The remainder of the paper is organized as follows. Section 2 presents a brief review of the extraction of indoor scene components and their spatial relationships. Section 3 gives the overview of the proposed method. Section 4 describes how to detect indoor scene components on the base of clustering of profiles. Section 5 elaborates on the inferring of spatial relationships between indoor scene components. The experimental results are presented in Section 6. The limitations of our method and proposals for future research are indicated in the last section.

## 2. Related Work

### 2.1. Detection of Indoor Scene Components

The extraction of indoor scene components from point clouds has received a lot of research interest in numerous works. Here we review the works of indoor scene components detection methods as follows. The methods can largely be classified into four types, i.e., the primitive shape proximity-based methods, the voxel grid-based methods, the data-driven methods, and other methods.

The primitive shape proximity-based methods approximate the indoor scene components with primitive shapes and use the primitive shape segmentation algorithms to detect indoor components. Rchnabel et al. [7] represented 3D semantic entities with configurations of basic shapes. Wang et al. [6] abstracted the sub-scenes with geometric primitives and their topological relationships with structural attributes. Li et al. [10] operated simultaneously on both the local and global aspects by fitting primitives locally while optimizing global relations iteratively. Hashemifar et al. [12] adapted a cuboid fitting algorithm for the mapping of indoor scenes. A limitation of the primitive shape proximity-based methods is that the commonly used primitive shape segmentation algorithms such as HOUGH transforming [13,14] and Random Sample Consensus (RANSAC) methods [15,16] are all based on statistical techniques. The segmentation results of these algorithms are randomly generated and the topological relationships between indoor scene components will be lost. It hinders the following spatial relationship analysis between the components. Most importantly, the shapes of indoor scene components are complex and varied. When using these methods to detect the components, segments of indoor scenes (i.e., components) with complex shapes will not be detected effectively. 

The voxel grid-based methods always adopt the 3D voxel grids representation of point clouds and perform segmentation on the simplified data structure. In [17], a point cloud was first voxelized by the octree. Then a K-means clustering algorithm was employed to realize super-voxel segmentation. Xu et al. [18] reported a novel strategy for segmenting 3D point clouds using a voxel structure and graph-based clustering with perceptual grouping laws. Lin et al. [19] proposed a new practice in super-voxel generation that adopted an adaptive resolution to preserve boundaries. It is challenging to accurately segment out components and preserve the boundaries in this kind of method.

The data-driven approaches often detect indoor scene components by training and applying a classifier to label the segments of indoor point clouds. For instance, Hausman et al. [21] pre-segmented a raw point cloud of a given scene using a part graph-based hashing algorithm, then an SVM-based classifier was trained by GRSD (Global Radius-based Surface Descriptor) feature and applied for the segments of point clouds. Recently, a few methods have employed deep learning to operate on indoor point clouds to segment out indoor scene components. Wang et al. [48] partitioned each object into smaller super-faces and each such super-face was associated with a vector of shape descriptors. Then must-link or cannot-link constraints between super-faces were added between super-faces through an active learning method. Qi et al. [22] designed a novel type of neural network (PointNet) that consistsedof a Classification Network and Segmentation Network. PointNet well respects the permutation invariance of points in the input and can be directly used for indoor scene components segmentation from point clouds. Li et al. [23] introduced a new deep learning-based method to parse 3D objects into moving parts based on input static shape snapshots. Te et al. [25] used a regularized graph convolutional neural network (RGCNN) for the semantic segmentation of object parts. However, the data-driven methods need to label the amounts of the point clouds scanned from indoor scenes, which is tedious work. In addition, these methods can only extract indoor scene components that observe comments in the training set and cannot discover new components. 

Researchers have also detected the indoor scene components by other methods. Stein et al. [31] de-composed the scene into an adjacency-graph of surface patches, where edges in the graph were classified as either convex or concave. Then the locally convex connected sub-graphs were extracted as components of indoor objects. Kaick et al. [32] presented a segmentation method for components with complete and incomplete shapes where the shape was first decomposed into approximate convex parts, then these were merged into consistent components based on a non-local geometric signature. Due to poor connectivity caused by missing parts and outliers of point clouds, the methods are not suitable for detecting indoor scene components from point clouds, especially for components with complex shapes. 

We propose a learning-free method that mainly exploits the similarity and spatial proximity of the profiles of slices of indoor scene components. There are some similar slicing-based methods [45,46,47,49,50] proposed to detect components of individual objects. Differently from these methods, our method mainly detects components of the whole indoor scenes. The segmentation of the indoor scene is more complex than that of the individual objects. By our method, the complex-shaped indoor scene components (from different individual objects) can be effectively extracted. 

### 2.2. The Acquisition of Spatial Relationships 

Extracting spatial relationships is crucial for the understanding of indoor scenes. There are many efforts that have been expended on the extraction of spatial relationships from scene images. Muda [33] used region boundaries and region labels to generate annotations describing absolute object positions and also relative positions between pairs of objects on the base of a domain ontology and spatial information ontology. Aditya et al. [34] presented a general architecture where the generic visual recognition techniques for the image scenes were implemented. Then a mapping between scene categories and inferred scene constituents was collected and implemented to predict relationships between scene constituents. Xu et al. [35] proposed a novel end to end model that solved the scene graph inference problem using standard Recurrent Neural Networks (RNNs) and learnt to iteratively improve predictions on objects and their relationships via message passing. In contrast with the spatial relationships in 2D images, the spatial relationships in the 3D point clouds are far more complex and the extraction of them is challenging.

Existing methods for the acquisition of spatial relationships directly from 3D point clouds can be divided into two categories, i.e., the machine learning-based methods and the knowledge-based methods. The machine learning-based methods mainly predefine the types of spatial relations and then train a classifier to predict the spatial relations. Silberman [38] introduced a principled approach that integrated physical constraints and statistical priors on support relationships to reason spatial semantics such as support from back, support from below, etc. Choi et al. [39] introduced a 3D Geometric Phrase Model (3DGP) which defined a group of object types (e.g., sofa, chair, table, etc.) and their 3D spatial configuration and proposed a latent SVM method to learn the interactions among scene objects. Because spatial relations are complex, it is difficult to establish a fixed parametric model for training. Thereby, the deep learning technology has recently been adopted by some researchers. Wald et al. [40] proposed two PointNet architectures for the extraction of objects and their spatial relationships and exploited a Graph Convolutional Networks to process the acquired object–object relationships. Although the deep learning techniques have shown prospects in the extraction of certain spatial relations, obtaining effective features of the complex spatial relationships is still difficult. 

The knowledge-based methods mainly use prior spatial knowledge to infer spatial relationships. Zender et al. [42] used an innate conceptual ontology that defined abstract categories for rooms and objects and how they are related to create conceptual map representations of human-made environments to represent spatial properties of typical indoor environments. Suchan and Bhatt [43] proposed an ontological characterization of human activities to extract commonsense spatio-temporal relations and patterns (e.g., left-of, touching, part-of, during, approaching, etc.) to offer human-centered automated reasoning about embodied spatio-temporal interactions with indoor environments. Ponciano et al. [44] proposed a knowledge system to detect the specific components of indoor objects and interleaved between spatial semantics inference and object recognition some spatial relationships (e.g., around, parallel) that had a close connection with recognition of indoor objects were extracted. In the context of spatial semantics extraction on synthetic data, Kontakis et al. [41] mimicked human spatial cognition and presented a knowledge-based index mechanism for the automated spatial correlation between objects in terms of linguistic predicts. Exiting knowledge-based methods mostly handle the spatial relationships between indoor objects. In our work, we use the spatial knowledge to infer the spatial relationships between indoor scene components from 3D point clouds. By introducing a slice strategy, the acquisition of spatial relationships between indoor scene components is facilitated.

## 3. Overview

**Figure 1:**
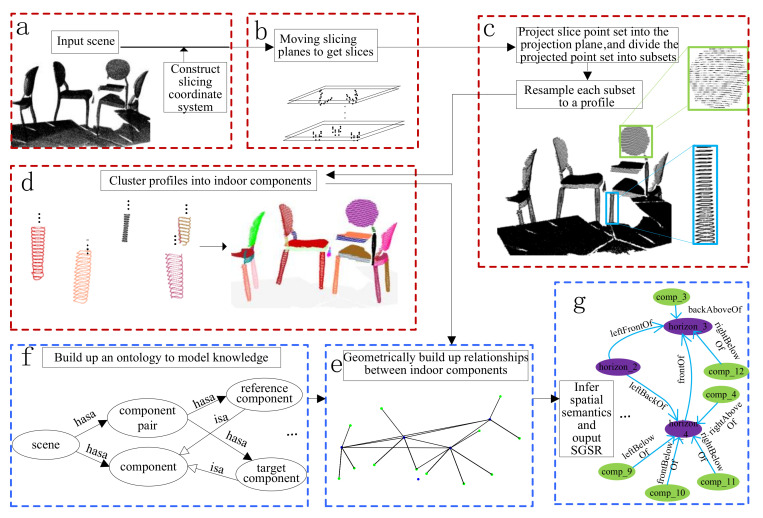
The framework of the proposed method: (**a**) Input an indoor scene model; (**b**) Slicing the indoor scene model; (**c**) Generate the profiles; (**d**) Cluster profiles into different indoor scene components; (**e**) Construct the relationship graph; (**f**) Build up an ontology to model knowledge about indoor scene components; (**g**) Infer spatial semantics and output SGSR of the input scene model.

Our work consists of two stages, i.e., the detection of indoor components and the spatial relationships inference. The framework is shown in Figure 1.

(1) Detection of indoor scene components
  (a) Given an indoor scene model, we firstly adopt a simple direction searching strategy to label the ground. Then we construct a slicing coordinate system (see Figure 1a), where the center of the bounding rectangle of the ground is taken as the origin, and the upward normal of the ground is taken as the *z*-axis. The *x*- and *y*-axes are chosen from two arbitrary orthogonal axes on the ground.
  (b) In the slicing coordinate system, from bottom to up, we iteratively slice the indoor scene model using two planes by a step size *h* in the perpendicular direction to the *z*-axis (see Figure 1b).
  (c) We project the point set of a slice on a projection plane and divide the projected point set into many subsets. Then each subset is resampled to a profile (see Figure 1c).
  (d) Profiles on different projection planes are clustered into indoor scene components based on the similarity and spatial proximity (see Figure 1c,d).
(2) Spatial relationships inference
  (a) We geometrically build up relationships between indoor scene components (see Figure 1e).
  (b) An ontology is constructed to model the commonsense knowledge about the semantics of spatial relationships between indoor scene components (see Figure 1f). Then the ontology is populated by the geometrically correlated indoor scene components.
  (c) The pair-wise spatial relationships are inferred by SWRL rules. An SGSR of the indoor scene model is output to represent the indoor scene components and their spatial relationships (see Figure 1g).

## 4. Detection of Indoor Scene Components

### 4.1. Slicing and Resampling of Indoor Point Clouds

To slice the indoor scene model effectively, the slicing coordinate system is constructed, where the center of the bounding box of the ground is taken as the origin. The upward normal of the ground is selected as the *z*-axis, and two arbitrary orthogonal axes on the ground are chosen as *x*-axis and *y*-axis. 

It is observed that most indoor objects are placed upright on the ground. Therefore, the ground can be labeled through a simple direction searching. The specific process is as follows. (1) Compute the Orientation Bounding Boxes (*OBB*s) of the indoor scene model and obtain outer planes $\Pi_{i}$, *i* = 0,1,2 … m (m ≤ 5) that correspond with the ground, the walls, and the ceiling. (2) Filter the points belonging to outer planes and segment the indoor scene model PC into point sets $PC=\cup_{i}\;(P_{i})$ by a k-nearest-neighbor (KNN) algorithm. (3) We select $\Pi_{i}$, *i* = 0,1,2 … m (m ≤ 5) as the ground and roughly regard each point set *Pi* as an object and generate *OBB*s from the resulting point sets. Moreover, due to the assumption that most objects are parallel to the ground, we enforce this constraint for the *OBB* computation—the orientation along the parallel plane of the plane $\Pi_{i}$. (4) If a $\Pi_{i}$ has the largest number of *OBB*s closest to itself, it is identified as the ground.

Motivated by the aim of ensuring that enough geometric features are included in each slice, we characterize the indoor scene slice as an indoor scene section with a thickness of *l*. The thickness *l* is computed by $\lambda_{d}d_{dens}$, $d_{dens}$ denotes the density of the point clouds, $\lambda_{d}$ is a density factor. $d_{dens}$ is formulated as the following equation,

(1) $$d_{dens}=\frac{1}{N}\sum_{i=1}^{N}\frac{1}{k}(\sum_{k=1}^{K}\left\|p_{i}-p_{k}\right\|)$$

where $p_{i},i=1,...,N$ denote a point of indoor point clouds, *p_k_* is the k-closest point of *p_i_*. *K* is set to 6.

**Figure 2:**
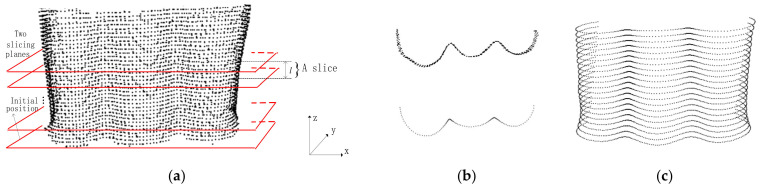
Slicing and resampling of point clouds: (**a**) Slicing of point clouds; (**b**) A slice and a profile; (**c**) Profiles of the point clouds.

The slicing position is initialized at the point that has the minimum *z*-axis value in the slicing coordinate system. Starting from the initial slicing position (the lower slicing plane is located at the initial position), from bottom to up, we iteratively slice the input indoor point clouds using two slicing planes by a step size *h* in the slicing direction, as seen in Figure 2. 

For each slice, a plane parallel to the slicing planes and located between the two slicing planes and equidistant from the two slicing planes is defined as the projection plane. On this basis, the point set of each slice is projected to the projection plane by setting the *z*-axis value of each point to the *z*-axis value of the intersection point of the projection plane and *z*-axis.

The projected point set of a slice is first divided into some subsets by the clustering algorithm [51]. Then each subset is thinned using the Moving Least Squire (MLS) method [52], and is thereafter resampled to a profile with an interval *d*. The size of *d* is calculated as $d=\frac{1}{N}\sum_{i=1}^{N}\frac{1}{K}\sum_{k=1}^{K}\left\|p_{i}-p_{k}\right\|$, where $p_{i}$ is a point of indoor point clouds, *p_k_* is the *k*-closest point of $p_{i}$. *K* is set to 5. Figure 2b shows one of slices of the object and the resampled point set, i.e., the profile of the slice. Figure 2c shows all the profiles of an indoor object.

Note that some special subsets do not need to be resampled. We divide the minimum bounding box (*MBB*) of each subset into many sub-rectangles and label the sub-rectangles that include one or more projected points, then count the labeled sub-rectangles and total sub-rectangles. If the ratio of a labeled sub-rectangles number to total sub-rectangles number is bigger than 0.7, the subset does not need to be resampled. More details about dividing *MBB* into sub-rectangles can be seen in [49]. The special subsets directly constitute a special kind of component of the indoor scene model. We refer to them as horizontal plane components (horizontal planes for short).

**Figure 3:**
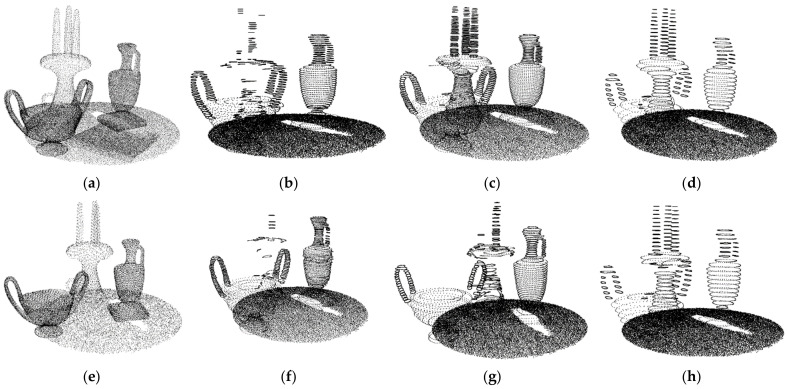

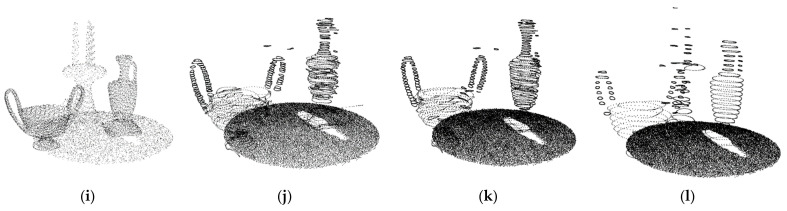
Experimental results of *l*: (**a**) Scene 1 (99538 points), $d_{dens}$ = 1.431; (**b**) $\lambda_{d}$ = 0.14, *l* = $\lambda_{d}$ $d_{dens}$; (**c**) $\lambda_{d}$ = 0.23, *l* = $\lambda_{d}$ $d_{dens}$; (**d**) $\lambda_{d}$ = 0.34, *l* = $\lambda_{d}$ $d_{dens}$; (**e**) 50% down-sampling model, $d_{dens}$ = 1.995; (**f**) $\lambda_{d}$ = 0.14, *l* = $\lambda_{d}$ $d_{dens}$; (**g**) $\lambda_{d}$ = 0.23, *l* = $\lambda_{d}$ $d_{dens}$; (**h**) $\lambda_{d}$ = 0.34, *l* = $\lambda_{d}$ $d_{dens}$; (**i**) 25% down-sampling model, $d_{dens}$ = 2.779; (**j**) $\lambda_{d}$ = 0.14, *l* = $\lambda_{d}$ $d_{dens}$; (**k**) $\lambda_{d}$ = 0.23, *l* = $\lambda_{d}$ $d_{dens}$; (**l**) $\lambda_{d}$ = 0.34, *l* = $\lambda_{d}$ $d_{dens}$.

To obtain the appropriate value of the density factor $\lambda_{d}$, we performed experiments on indoor scene models with different densities. By using different sampling rates to down-sample the indoor point cloud, point clouds with different densities can be obtained. Given a tabletop scene model (Figure 3a), we chose the original model, the 50% down-sampling model, and the 25% down-sampling model for the experiments. We set $\lambda_{d}$ to 0.14, 0.23, and 0.34 for the models. The results are shown in Figure 3b–l). If $\lambda_{d}$ is smaller, the slice will be thinner. The thinner the slice, the fewer points on the slice. In a severe case, profiles will fail to be obtained. As can be seen, the profiles are largely missing when $\lambda_{d}$ is 0.14 or 0.23. When $\lambda_{d}$ is 0.34, good results are achieved. In our work, $\lambda_{d}$ was set to 0.34.

**Figure 4:**
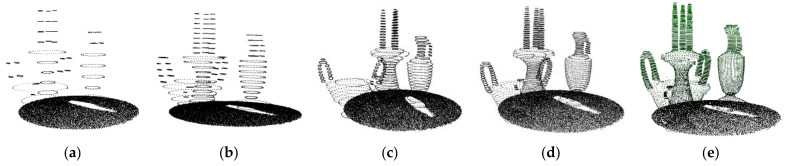
Experimental results of *h*: (**a**) *l* = 0.34 $d_{dens}$, *h* = 4 *l*; (**b**) *l* = 0.34 $d_{dens}$, *h* = 2 *l*; (**c**) *l* = 0.34 $d_{dens}$, *h* = 1.0 *l*; (**d**) *l* = 0.34 $d_{dens}$, *h* = 0.8 *l*; (**e**) *l* = 0.34 $d_{dens}$, *h* = 0.4 *l*.

*h* depends on the expected number of slices. *h* will affect the running time. The smaller the *h*, the longer the cutting and resampling process will take, as shown in Table 1. Different slicing results of the scene are shown in Figure 4. *H* was finally set to 1.0 *l*.

### 4.2. Clustering of Profiles

Let the total resampled point set of indoor scene model be ℜ, and let each profile be $\lambda_{ij}$ (i.e., the *j*th profile of the *i*th slice), then ℜ = ${\left\{{\left\{\lambda_{ij}\right\}}_{j=1}^{M_{i}}\right\}}_{i=1}^{N}$.

Given two profiles $\lambda_{ij}$ and $\lambda_{qk}$ of ℜ, their spatial proximity and similarity are evaluated. To judge whether two profiles are adjacent, their *MBB*s are calculated and denoted as *MBB*_1_ and *MBB*_2_, respectively. If $\lambda_{ij}$ and $\lambda_{qk}$ belong to neighboring slices and *MBB*_1_ and *MBB*_2_ are overlapped, $\lambda_{ij}$ and $\lambda_{qk}$ have spatial proximity. 

For two profiles with spatial proximity, their similarity is further judged. A similarity measure $D_{sc}(\lambda_{ij},\lambda_{qk})+(1-\min(\frac{MBB_{1}}{MBB_{2}},\frac{MBB_{2}}{MBB_{1}}))$ is designed, where $D_{sc}(\lambda_{ij},\lambda_{qk})$ computes the distance between shape context features [53] of the two profiles, $\min(\frac{MBB_{1}}{MBB_{2}},\frac{MBB_{2}}{MBB_{1}})$ is adopted to approximate the scale ratio of the two profiles. If the similarity measurement between $\lambda_{ij}$ and $\lambda_{qk}$ is smaller than a threshold δ, the two profile $\lambda_{ij}$ and $\lambda_{qk}$ belong to the same component. Starting from the initial profiles, the profile pair in ℜ are iteratively clustered into different components, i.e., the profile clustering-based components. 

**Figure 5:**
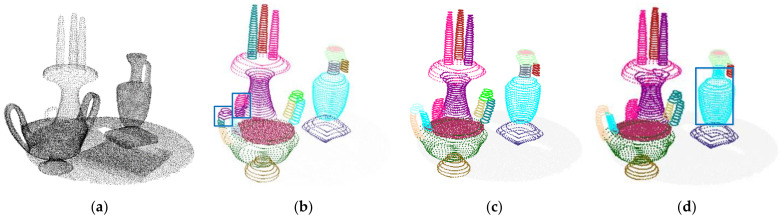
Experimental results of δ: (**a**) Scene 1; (**b**) δ = 0.38; (**c**) δ = 0.48; (**d**) δ = 0.58.

To evaluate the effect of threshold δ on the clustering results, we set δ to 0.38, 0.48, 0.58, respectively, and the clustering results are shown in Figure 5. It can be seen that the smaller δ may result in over-segmentation (see the blue rectangle in Figure 5b), and the bigger δ may result in under-segmentation (see the blue rectangle in Figure 5d). We set δ to 0.48 in our work.

**Figure 6:**
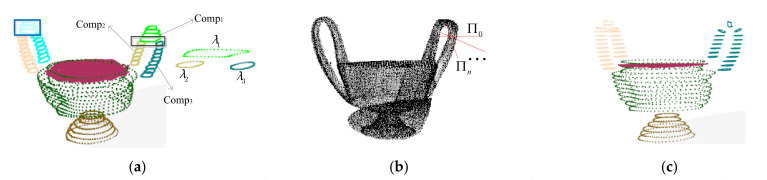
Adjustment of slicing: (**a**) The initial segmentation result; (**b**) rotated slicing planes; (**c**) the final results.

Note that some complex-shaped components may be over-segmented due to the profiles in some local surfaces of the components (see the blue rectangle in Figure 6a). To solve the problem, we will locally adjust the slicing direction in a way similar to the method in [45] at this local surface. Specifically, given three components, *Comp*_1_, *Comp*_2_, *Comp*_3_, if a profile of *Comp*_1_ is respectively overlapped with its neighbor’s profiles that belong to *Comp*_2_ and *Comp*_3_ (see Figure 6a), we label the component set as ${\left\{Comp_{1},Comp_{2},Comp_{3}\right\}}_{c}$, and we reslice the raw points that correspond with *Comp*_1_. A rotational slicing direction will iteratively be applied in the raw points (see Figure 6b) until the optimal slicing is found. Then we re-cluster the re-generated profiles and the profiles of *Comp*_2_ and profiles of *Comp*_3_, and update the clustering results (see Figure 6c) according to the minimum number of components principle.

**Algorithm 1. ** Clustering profiles into indoor scene components.**Input:**ℜ = ${\left\{{\left\{\lambda_{ij}\right\}}_{j=1}^{M_{i}}\right\}}_{i=1}^{N}$, $\lambda_{ij}$ is the *j*th profile on ith projection plane, δ
**Output:** {*Comp_l_*}1.*l* = 0;2. *for i* = 1:1:*N*3.  *for j* = 1:1:*M_i_ do*
*Comp_l_*
←ϕ;4.   *if*
$\lambda_{ij}$ is not marked5.    $\lambda_{s}$←$\lambda_{ij}$, mark $\lambda_{ij}$;//6.     *u* = *I* + 1; *do* Γ←${\left\{\lambda_{uv}\right\}}_{v=1}^{M_{u}}$ & $\lambda_{uv}$ is not marked; Γ←ϕ;7.      search spatial&similar profile $\lambda_{r}$ of $\lambda_{s}$ in Γ;8.      *Comp_l_*
← *Comp_l_*
∪$\lambda_{r}$; mark $\lambda_{r}$; $\lambda_{s}$←$\lambda_{r}$;9.      *u* = *u* + 1;10.     *until*
$\lambda_{r}$ is not found11.   *l* = *l* + 1; end *if*12.  end *for*13. end *for*14.  Search component set ${\left\{Comp_{p},Comp_{q},Comp_{k}\right\}}_{c}$ from {*Comp_l_*}, *p*, *q*, *k* ∈[0,*l*], 15. *for* each ${\left\{Comp_{p},Comp_{q},Comp_{k}\right\}}_{c}$
16.   apply rational-direction slice in raw points that correspoing with *Comp_p_*
17.  re-genarate profiles;18.   re-cluster the profiles and profiles of *Comp_q_*, *Comp_k_*, and update {*Comp_l_*}19. end *for*20. output {*Comp_l_*}

## 5. Spatial Semantics Inference

### 5.1. Spatial Relationships 

#### 5.1.1. Topological Relationships 

Topology is a fundamental aspect of space. There are some popular formalizations of topological relations in 3D space. Region Connection Calculus (RCC) [36] is a popular formalization of topological relationships. It provides occlusion support by considering the projections of 3D objects in 2D space. In [37], the Dimensionally Extended Nine-Intersection Model (DE-9IM) defined 11 types of topological relationship such as disjoint, meet, contains, covers, inside, covered by, equal, etc., according to the boundary-based intersection pattern between two 3D objects. 

In our work, we mainly focus on two kinds of topological relationships between indoor scene components, i.e., connect, adjacent. They are defined on the basis of two geometric distances. The first distance is the minimal distance between two components. It is calculated as $d_{1}(A,B)=\inf_{p_{1}\in A,p_{2}\in B}d(p_{1},p_{2})$, where *A* and *B* are components, *p*_1_ are *p*_2_ are two points that belong to *A* and *B*, respectively. The second distance is the distance between the centroids of two components. It is calculated as $d_{2}(A,B)=d(centroid(A),centroid(B))$. 

Because indoor scene components have different scales, e.g., the components of furniture and the components of tabletop objects, we adopt the following principles for building up topological relationships between two indoor scene components. 

(1) We divide the indoor scene components into two categories, i.e., *Class_I* and *Class_II*, according to their scales. Specifically, if the area of a horizontal plane is bigger than the area threshold, or the volume of a profile clustering-based component is bigger than the volume threshold, the component is classified into *Class_I* components. Otherwise, the component is classified into *Class_II* components. 

(2) For two *Class_I* components *A* and *B*, if $d_{1}(A,B)<\sigma_{1}$, they are connected. If $d_{1}(A,B)>\sigma_{1}$ and $d_{2}(A,B)<\sigma_{2}$, they are adjacent. In our work, $\sigma_{1}$ was set to 0.35, $\sigma_{2}$ was set to 3.

(3) For two *Class_II* components *A* and *B*. if $d_{1}(A,B)<\sigma_{1}$, they are connected. If $d_{1}(A,B)>\sigma_{1}$ and $d_{2}(A,B)<\sigma_{2}$, they are adjacent. $\sigma_{1}$ was set to the same value as that of *Class_I* component. $\sigma_{2}$ was set to 0.75.

(4) For a *Class_I* horizontal plane and a *Class_II* component, we will judge whether they are connected or not. The adjacent relationships between them are not considered. Moreover, we only consider the connected relationship between the *Class_I* horizontal plane and the *Class_II* components. The topological relationships between the other *Class_I* components and *Class_II* components are not considered. 

#### 5.1.2. Directional Relationships

**Figure 7:**
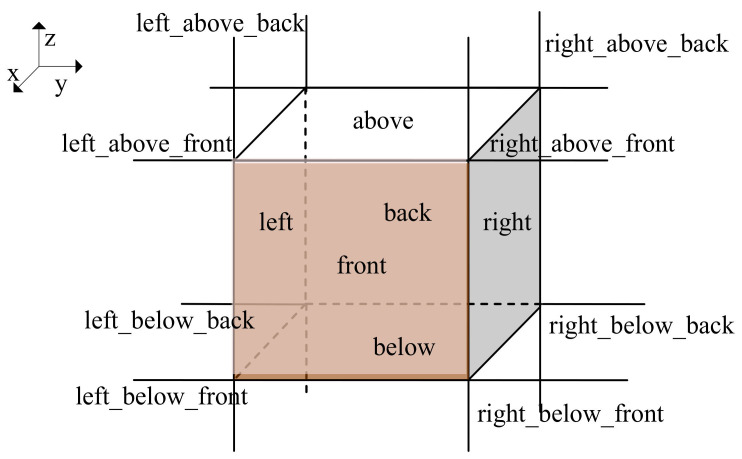
Directional relationships.

Directional relations refer to another major category of spatial analysis. The directional relationships mainly describe the relative position of 3D components in a coordinate system. In order to depict the directional relationships between the indoor components finely, we divide 3D space around an indoor scene component into 14 sub-spaces, i.e., above, below, left, right, front, back, left_above_back, left_above_front, etc., as seen in Figure 7. 

Obviously, given a reference component *B*, when a component *A* is located in the six subspaces around the reference component, i.e., above, below, left, right, front, and back, there at exists least six corresponding directional relationships between *A* and *B*, i.e., leftOf, rightOf, frontOf, backOf, ect. We take the leftOf or rightOf as the examples to illustrate how to decide the directional relationships. The slicing coordinate system is taken as the reference coordinate system. The directional relationship of indoor scene components is determined through the coordinates of vertices of the indoor scene component’s *MBB*s. 

**Figure 8:**
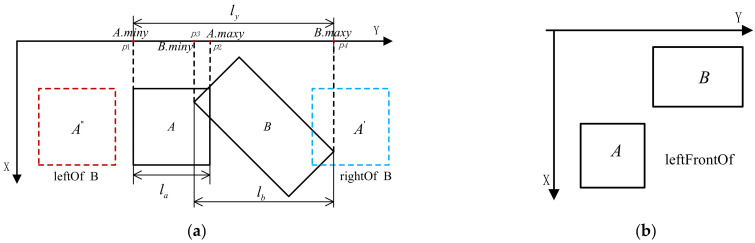
Directional relationships: (**a**) The leftOf and rightOf relationships; (**b**) the leftFrontOf relationships.

We first project their *MBB* vertices that have maximum and minimum *y*-axis coordinate value onto the *y*-axis, as shown in Figure 8. Then we calculate the length of the longest line segment formed by the projected vertices, i.e., $l_{y}$. Next, for *MBB* of *A* or *B*, we calculate the distance between the vertices that respectively have the largest and the smallest *y*-axis coordinate value, i.e., $l_{a}$ and $l_{b}$. Let $l_{yo}=l_{a}+l_{b}$, if $l_{y}/l_{yo}$ is larger than 0.8, and *A_MBB*.maxy ≥ *B_MBB*.maxy, then *A* is in the right of *B* (see the blue rectangle *A*), as seen in Equation (2). On the contrary, *A* is in the left of *B* (see the red rectangle *A*), as shown in Equation (3).

(2) $$isRightOf(A,B)\leftarrow \;l_{y}/l_{yo}\geq \tau \;\&\&\;A\_MBB.maxy\geq B\_MBB.maxy$$

(3) $$isLeftOf(A,B)\leftarrow \;l_{y}/l_{yo}\geq \tau \;\&\&\;A\_MBB.miny<B\_MBB.miny$$

In some cases, there may be two directional relationships between component *B* and component *A* simultaneously. For example, if component *A* is located at the front-left of component *B*, as seen in Figure 8b. This two spatial relations are collectively denoted as *isleftFrontOf* (*A*,*B*). It depends on the projection of their *MBB*s on *x*-axis and *y*-axis. There also are some similar directional relationships, e.g., *leftFrontOf* (*A*,*B*), *rightFrontOf* (*A*,*B*), etc.

If component *A* is located in the eight sub-spaces around the reference component *B* such as left_above_back, left_above_front, etc., there will be corresponding relationships *leftFrontAboveOf* (*A*,*B*), and *rightFrontOf* (*A*,*B*), etc., between them. Similarly, these directional relationships are defined on the *x*-axis, *y*-axis, and *z*-axis coordinates. 

### 5.2. Ontology-Based Spatial Semantics Inference

**Figure 9:**
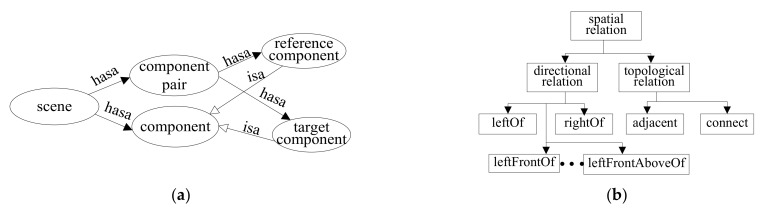
The scene ontology: (**a**) The scene ontology; (**b**) the spatial relationships.

We built up an ontology to model the common sense knowledge about the semantics of spatial relationships between indoor scene components, as seen in Figure 9a. The ontology consists of a number of concepts arranged hierarchically. The root concept is a scene concept with two sub-concepts, i.e., component and component pair. The component pair concept includes two sub-concepts, i.e., the reference component and the target component. The hierarchical spatial relationship between indoor scene components is shown in Figure 9b. The properties of the ontology concepts are enumerated in Table 2. 

We firstly geometrically built relationships between indoor scene components on the base of two geometric distance *d*_1_ and *d*_2_ and obtain component pairs. Then the ontology concepts are populated by a related component. For each component pair instance, the horizontal plane is preferred as the reference component, and the profile clustering-based component is preferred as the target component. If both components are horizontal planes or the profile clustering-based components, any one of them is instantiated as the reference component, and the other one is instantiated as the target component. 

We mainly adopt Semantic Web Rule Language (SWRL) to regulate rules for inferring spatial semantics. For instance, the following assert the topological relationships and spatial relationships between two components, respectively.
*isAdjacent*(?*A*,?*B*)→*isComPair*(?*comPair*)^*hasReferenceComp*(?*comPair*, ?*A*) ^ *hasTargetComp* (?*comPair*, ?*B*) ^ *Distance1* (?*A*, ?*B*, ?*dis*_1_) ^ *swrlb*: *greaterThan*(?*dis*_1_, $\sigma_{1}$) ^ *Distance2*(?*A*,?*B*, ?*dis*_2_) ^ *swrlb*: *greaterThan*(?*dis*_2_, $\sigma_{2}$)*isLeftOf*(?*A*,?*B*)→*isComPair*(?*comPair*)^*hasReferenceComp*(?*comPair*, ?*A*) ^ h*asTargetComp* (?*comPair*, ?*B*) ^ *hasMBB* (?*A*,?*A_MBB*) ^ *hasMBB*(?*B*,?*B_MBB*) ^ ?*Length1*(?*A_MBB*,?*B_MBB*,?*ly*) ^ *Length2*(?*A_MBB*,? *B_MBB*,?*lyo*)^*MinY*(?*A_MBB*,? *A_MBB_miny*) ^*MinY*(?*B_MBB*,?*B_MBB_miny*)^*swrlb*: *greaterThan*(?$l_{y}$,$\tau l_{yo}$?) ^ *swrlb*: *smallerThan*(?*A_MBB_miny*,? *B_MBB_miny*)

**Figure 10:**
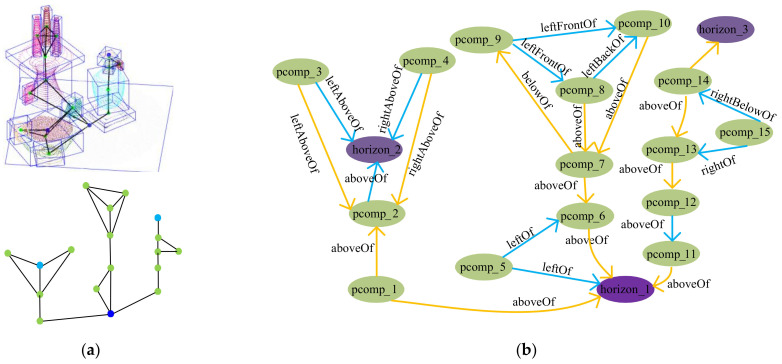
SGSR of a scene: (**a**) The tabletop scene and the relationships graph of scene; (**b**) SGSR of scene.

We adopt a graph to represent the indoor scene components and spatial relationships as seen in Figure 10a, where the light green nodes represent the *Class_II* profile clustering-based components, the light purple nodes represent the *Class_II* horizontal plane, the dark purple nodes represent the *Class_I* horizontal plane, a pair of nodes connected by edges represent two geometrically related components. The component in each component pair is taken as the node of SGSR. The inferred spatial relationships are added into SGSR as edges as shown in Figure 10b, where the blue edges denote the adjacent relationships, and the yellow edges denote connect relationships. To limit redundancy, the adjacent relationships between the components that share a horizontal plane have been filtered. The directional relationships whose reference component and target component have been exchanged each other have also not been shown.

## 6. Experiments

### 6.1. Evaluation of Indoor Scene Components Segmentation

#### 6.1.1. Experiments of Indoor Scene Components Detection

**Figure 11:**
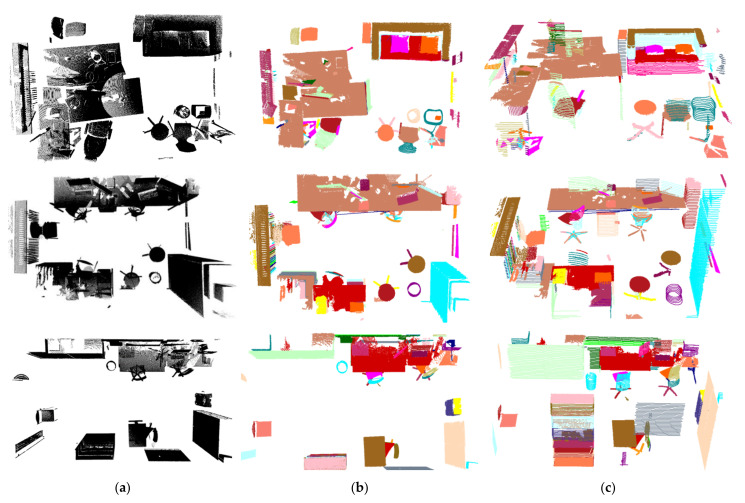
Experiment results of ETH scenes, different colors denote the components of indoor scenes: (**a**) Input scenes; (**b**) Top views of components of scenes; (**c**) Front views of components of scenes.

To evaluate the effectiveness of the proposed method, we ran an extensive set of experiments on some indoor scene models. The proposed algorithm was programmed with VC++ and OpenGL for display and rendering. All of the experiments in this paper were run on a PC with IntelI CoreI2, CPU2.80GHz, 2G memory. We evaluated our method on ETH [54], TUM [55] and dataset [56]. We empirically set $\lambda_{d}$ = 0.34, *h* = 1.0 × *l*, δ = 0.48 for the experimental scenes. ETH is a high-quality point cloud dataset containing 18 office scenes. It mainly includes indoor objects such as chair, desk, keyboard, monitor, mouse, cup, cabinet, lamp, sofa, pillow and so on. Clutter and occlusion were presented in the dataset. In order to show the scenes clearly, we removed the walls, ceilings and ground from the scenes. The qualitative experimental results are shown in Figure 11. They show that most of the components were detected correctly. Besides, some objects in the scenes were cuboid-like or cylinder-like (e.g., cabinets, boxes, and bottles). Each of them was simply structured and should have been an individual component of the indoor scene. However, considering that our proposed method can segment out the plane parallel to the slicing planes from each object, these objects were labeled as two components in the ground truth, i.e., the body and the cover (i.e., a plane parallel to the slicing planes). Although it is inconsistent with the general ground truth (i.e., these objects are individual components), this segmentation (i.e., label the body and the cover of the object) is still meaningful. 

**Figure 12:**
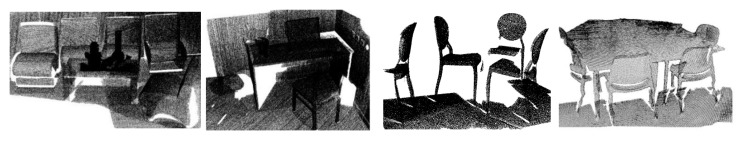

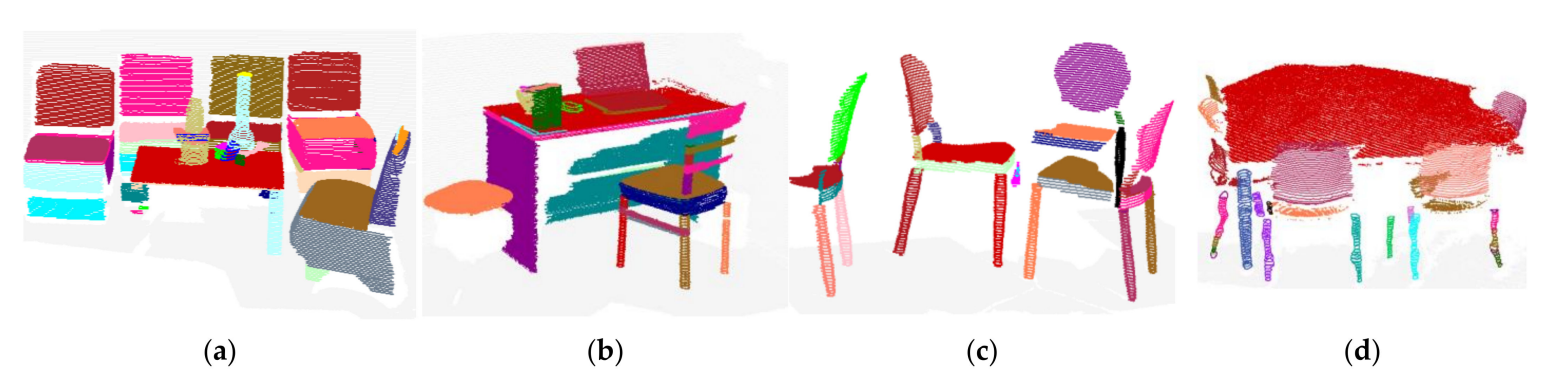
Experimental results of scenes of dataset [56], the first row is the input scene model, the second row is the indoor scene components detection result: (**a**) Living room; (**b**) Office; (**c**) Lounge; (**d**) Meeting room.

The dataset [56] included different kinds of scenes such as living rooms, office rooms, meeting rooms. Figure 12a–d respectively shows the detected indoor components of living room, office, lounge, and meeting room. It can be seen that most indoor scene components have been detected successfully, which is expected from the results presented in the previous section.

**Figure 13:**
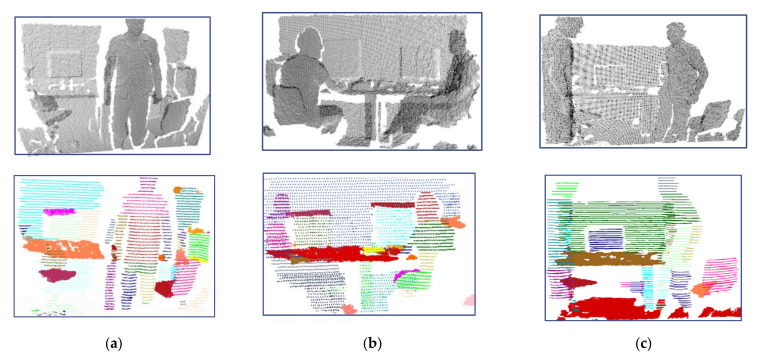
Experimental results of TUM scenes, the first row is the input scene model, the second row is the indoor scene components detection result: (**a**) Scene with a standing person; (**b**) Scene with two sitting person; (**c**) Scene with two standing person.

TUM dataset is a low-quality RGBD dataset and its scenes include persons. Generally speaking, persons have more complex shapes than furniture. Through the TUM dataset, the effectiveness of the proposed method for complex shaped indoor components was evaluated. Figure 13a–c respectively shows the detected indoor scene components of three kinds of scenes that including person with different poses. It can be seen that the walls, computer screens, table tops, most body parts of persons have been detected effectively. Moreover, the TUM dataset is made up of low-quality RGBD data, which increased the difficulty of detection of components. Due to the adoption of profile features instead of point features, the general object and human separation of our method was still very good. The executing time of our method on the three datasets are shown in Table 3.

**Figure 14:**
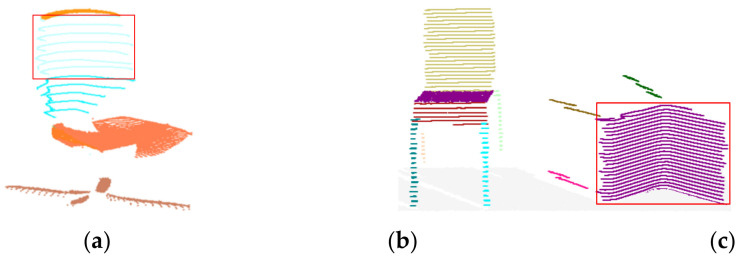
Limitations: (**a**) Objects with special shaped components; (**b**) Objects with normal posture; (**c**) Objects with abnormal posture.

A few special-shaped components of objects were over-segmented, e.g., the chair back in Figure 14a. However, the main part of the chair back (the red rectangle) was detected and preserved. A limitation of our method is the determination of slicing direction. We adopted a fixed slicing direction that was orthogonal with the normal direction of the ground. If some objects are not placed on the ground with an up-right posture, some components will not be detected correctly, as shown in Figure 14c.

#### 6.1.2. Comparison of the Methods

**Figure 15:**
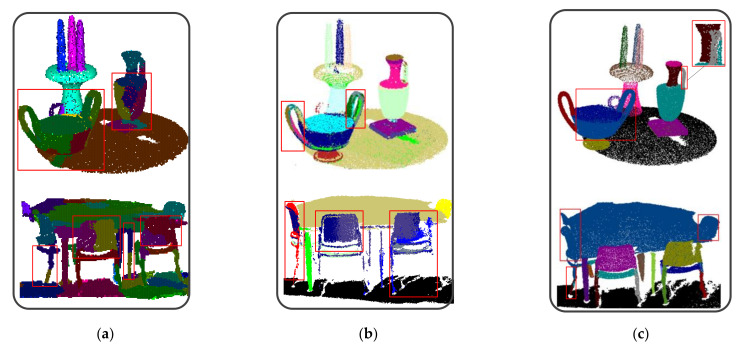

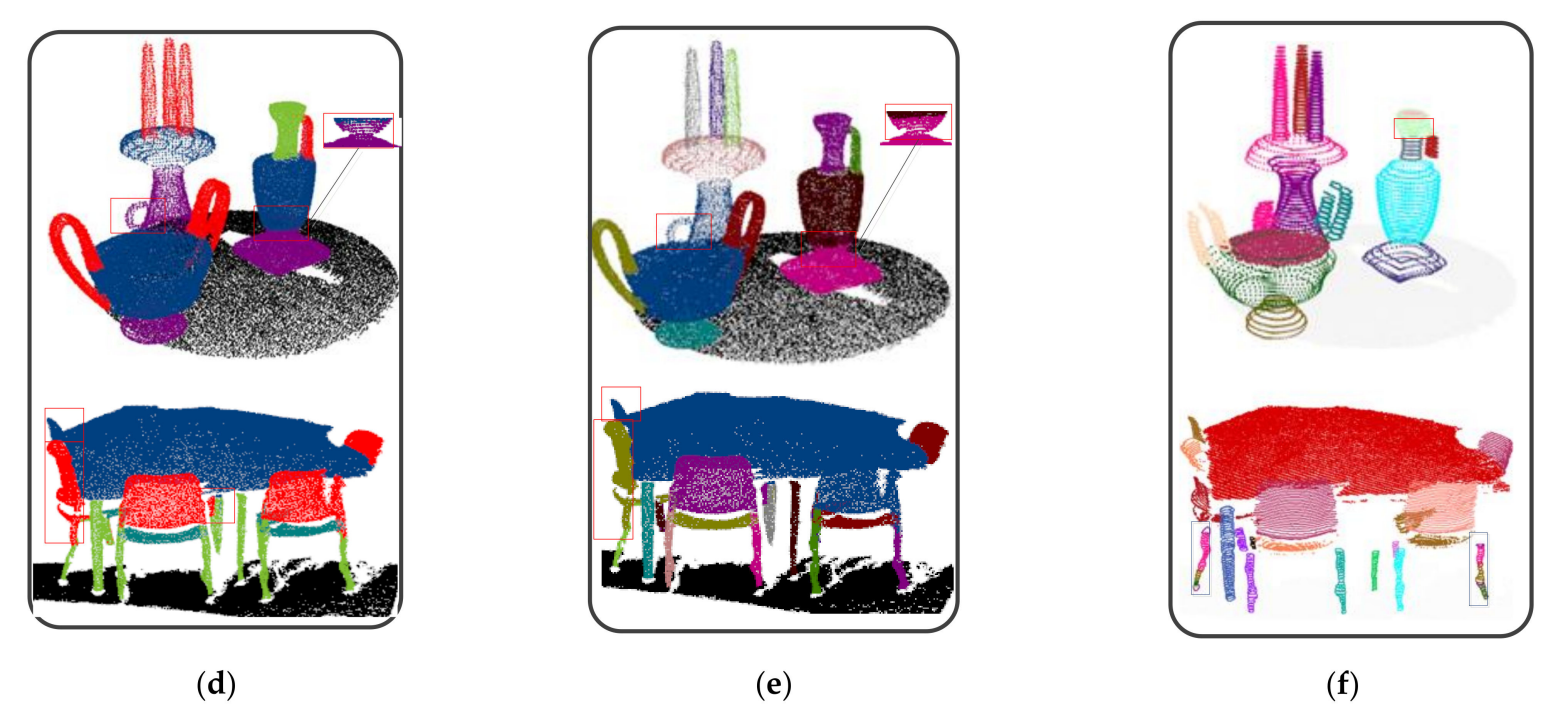
Components of the indoor scene models: (**a**) LCB; (**b**)RANSAC; (**c**) CRF; (**d**) Semantic segmentation result of PointNet++; (**e**) Instance segmentation result of PointNet++; (**f**) The proposed method.

To demonstrate the advantage of the proposed method, we compared our method with RANSAC [16], a local convexity-based method (LCB) [31], a CRF-based method [27] and PointNet++ [28] on the tabletop scene and the meeting room. The results are shown in Figure 15. It can be seen that the complex-shaped indoor scene components such as the bowl handle and some chairs and legs were not detected exactly by LCB and RANSAC. With the help of machine-learning technology, CRF achieved better results than LCB and RANSAC. However, CRF still failed to detect some components due to the fact that the optimal CRF model parameters are difficult to obtain. The semantic segmentation results of PointNet++ are shown in Figure 15d, where the components semantic categories are labeled. The indoor scene components with same semantic categories are further separated by a classification network and the instance components are shown in Figure 15e. A few of the components were not detected accurately by PointNet++ because of the wrong semantic label of points. 

The quantitative results of Figure 15 are shown in Table 4, where the totally detected components numbers (TN) and the correctly detected components numbers (RN) are counted. A detected indoor scene component was considered to be correctly detected if its IoU overlap ratio with the *MBB* of the ground truth components was larger than a threshold. IoU is the volume of the 3D intersection of the *MBB*s, divided by the volume of their 3D union. Here, the threshold overlap ratio was set to 0.7. The ground truth indoor scene components of the scenes were manually labeled, and the ground truth numbers (GN) of the tabletop scene and meeting room were17 and 20, respectively. Table 4 shows more of the components of the scenes correctly detected by the proposed method.

The quantitative results of the experiments are shown in Table 5. The extraction ratio and error ratio of indoor scene components was introduced to evaluate the methods. The extraction ratio was expressed as $ratio\_extra=\frac{RN}{GN}$. Moreover, the error extraction ratio was formulated as $ratio\_error=\frac{TN-RN}{TN}$. It shows that the proposed method had a higher extraction ratio than LCB and RANSAC. In comparison with CRF and PointNet++, the proposed method achieved a higher extraction ratio of the indoor scene components. However, because the proposed method may have generated more segments, its error extraction may have also been higher than CRF and PointNet++. In comparison with LCB, CRF, and PointNet++, the proposed method had a lower IoU overlap ratio with the ground truth. This was due to the over-segmentation of some special shapes. 

### 6.2. Evaluation of Spatial Semantics Acquisition 

**Figure 16:**
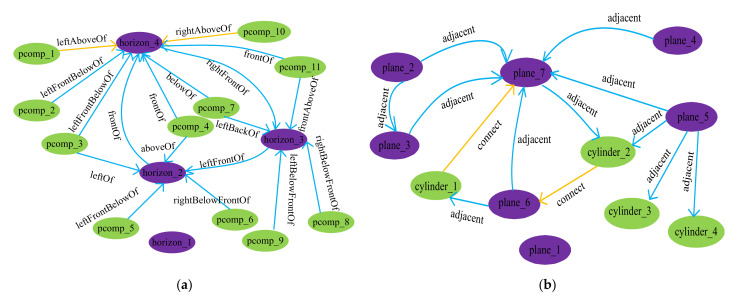
Spatial relationships predicts: (**a**) SGSR of the meeting room of Figure 15f; (**b**) Spatial relationships predicted on the base of the baseline [57].

Here, we report the 3D spatial semantics inference results of our method. We represent the component and the spatial relationships of the meeting room by SGSR, as seen in Figure 16a. We compared our method against a relationship prediction baseline inspired by baseline [57]. The baseline extracted indoor components from point clouds on the base of 3D primitive shapes approximation and built up the topological relationships (e.g., adjacent, parallel, and orthogonal, etc.), as seen in Figure 16b. We represented each spatial relationship as a triplet (*Comp_i_*, *relationship*, *Com_j_*) and compared the triplets of SGRS with the ground truth triplets. If *Comp_i_* and *Com_j_* of a triplet, respectively, had an IoU overlap ratio of 0.7 or higher with that of a ground truth triplet, and the relationship of the triplet was also the same with that of the ground truth triplet, the triplet of SGSR was considered as correctly predicted. The extraction rate was defined as the number of the correctly predict triplets against the number of the ground truth triplets. The triplets of generated inspired by baseline [57] were also compared with the ground truth in the same way. The extraction ratio of the proposed method and the baseline are shown in Table 5. It shows that SGSR represented the structure of indoor scenes more exactly. 

## 7. Conclusions

We present a framework to detect the complex shaped indoor components and infer their spatial relationships. The kernel is a slice-guided indoor scene components detection algorithm for indoor point clouds. The core insight is that slices of most components of indoor scenes always have similar 2D profiles, which allows for the detection of complex shaped components regardless of whether these components have regular geometry. Besides, through the layers of global slicing, the topological relationships between indoor components were reserved and the construction of spatial relationships between indoor components was also facilitated. 

To obtain a spatial structure of indoor scene models, we built up an ontology to model the commonsense knowledge about the semantics of spatial relationships between indoor scene components. The spatial relationships between indoor components were inferred and a SGSR was constructed to represent the components and their spatial relationships. 

With experimental evaluation, we demonstrated the segmentation performance of our proposed method on indoor scene components with complex shapes. We have also shown that our method can exactly predict spatial relationships.

A limitation of our method is the calculation of slicing direction. When using our proposed method, different slicing directions will lead to different segmentation results. In a real indoor scene, most objects are placed on the ground in a normal posture, thus the perpendicular direction to normal of floor is selected as the slicing direction and the segmentation results are satisfied. For the objects placed on the ground with an abnormal posture and the objects having special shapes, how to determine the slicing direction and how to detect the components are our future work.

## Figures and Tables

**Table 1:** Executing times.

*h*	0.4 *l*	0.8 *l*	1.0 *l*	2 *l*	4 *l*
times	450 s	285 s	240 s	180 s	126 s

**Table 2:** Properties of ontology concepts.

Properties of Component Concept	Meaning	Properties of Component Pair Concept	Meaning
hasType	horizontal plane or profile clustering-based component	hasReferenceComp	a relation starting from a component
hasArea	area of the horizontal plane	hasTargetComp	a relation target at a component
hasVolume	volume of *MBB* of profile clustering-based component		
hasMBB	*MBB* of the component		

**Table 3:** Mean executing times on dataset.

Dataset	ETH	Dataset [56]	TUM
Mean time	4300 s	2105 s	1100 s

**Table 4:** The quantitative results of Figure 15.

	LCB		RANSAC		CRF		PointNet++		Our Method	
	TN	RN	TN	RN	TN	RN	TN	RN	TN	RN
Tabletop scene	23	9	28	16	17	15	14	14	18	17
Meeting room	33	14	20	9	20	15	19	18	21	15

**Table 5:** The extraction ratio of indoor scene components and topological relationships.

	Components			Error Ratio of Components			Mean IoU Overlap	Topo-Relationships
	EHT	Dataset [56]	TUM	EHT	Dataset [56]	TUM		
LCB	0.60	0.55	0.49	0.56	0.64	0.66	0.92	-
RANSAC	0.66	0.65	0.70	0.43	0.50	0.41	0.86	-
CRF	0.86	0.84	-	0.17	0.18	-	0.90	-
Baseline [57]	-	-	-	-	-	-	-	0.72
Our method	0.86	0.87	0.88	0.23	0.20	0.18	0.87	0.83
PointNet++	0.87	0.84	-	0.16	0.15	-	0.89	-

## Data Availability

Publicly available datasets were analyzed in this study. This data can be found here: https://www.ifi.uzh.ch/en/vmml/research/datasets.html; https://3d.bk.tudelft.nl/liangliang/publications.html; https://vision.in.tum.de/data/datasets/rgbd-dataset/download (accessed on 14 December 2021).
